# Presentation of metastatic carotid body paraganglioma on F-18 FDG PET/CT: a rare disease

**DOI:** 10.1186/s41824-024-00211-x

**Published:** 2024-07-08

**Authors:** Mehul Dulet, Vaibhav Trivedi, Deepanksha Datta, Poonam Elhence, Rajesh Kumar

**Affiliations:** 1 Department of Nuclear Medicine, All India Institute of Medical Sciences, Jodhpur, India; 2 Department of Pathology and Lab Medicine, All India Institute of Medical Sciences, Jodhpur, India

**Keywords:** Malignant carotid body tumor, F-18 FDG PET/CT, Metastases, Paraganglioma

## Abstract

Carotid body paraganglioma is a slow growing tumor of head and neck region. It can rarely be malignant in nature which is characterized by distant metastases on anatomical imaging. We share an interesting presentation of a malignant carotid body on F-18 FDG PET/CT in form of liver and skeletal metastases.

## Introduction

Carotid body tumor is a slow growing neuroendocrine tumor arising from the paragangliomic cells (Barnes et al. 2005). It is the most common head and neck paraganglioma, and is usually benign in nature (Lack et al. 1977). The anatomical imaging using ultrasound—doppler neck, contrast enhanced computed tomography (CECT) and magnetic resonance imaging (CE-MRI) are the initial investigations of choice to detect this tumor. It is rarely malignant in nature, and functional imaging plays a crucial role in the staging as well as response assessment of this disease. Here, we share an interesting case of metastatic carotid body paraganglioma and its presentation on F-18 FDG PET/CT.

## Case report

**Fig. 1:**
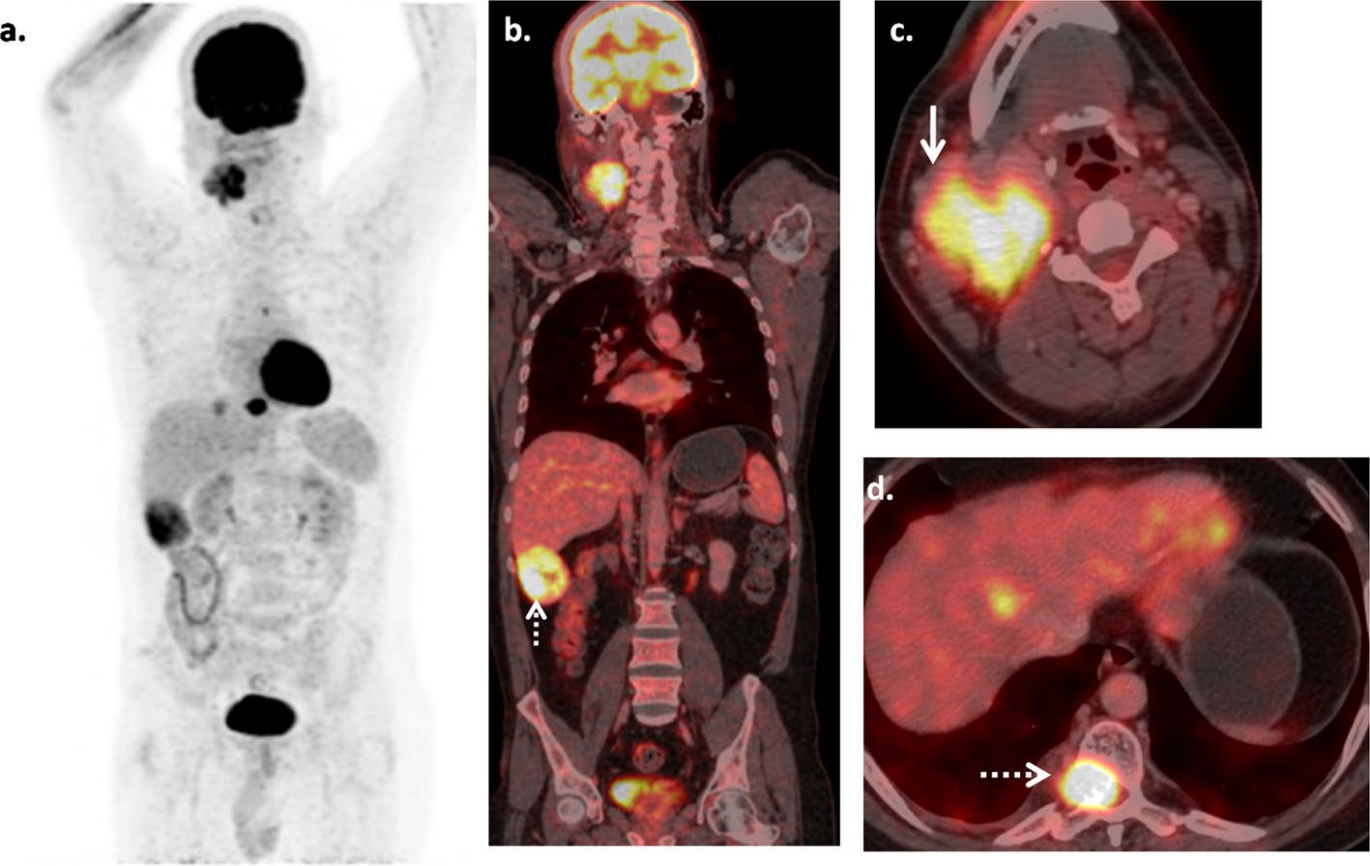
The Maximum intensity projection (**a**) and fused coronal PET/CT (**b**) and axial (**c**) images show abnormal FDG uptake in right carotid space causing splaying of the carotid arteries with their encasement (white arrows). Note is made of the metabolically active hepatic lesion in segment V–VI (dotted arrows) in fused coronal image (**b**) and lytic lesion in D9 vertebrae (dotted arrows) in fused axial image (**d**)

**Fig. 2:**
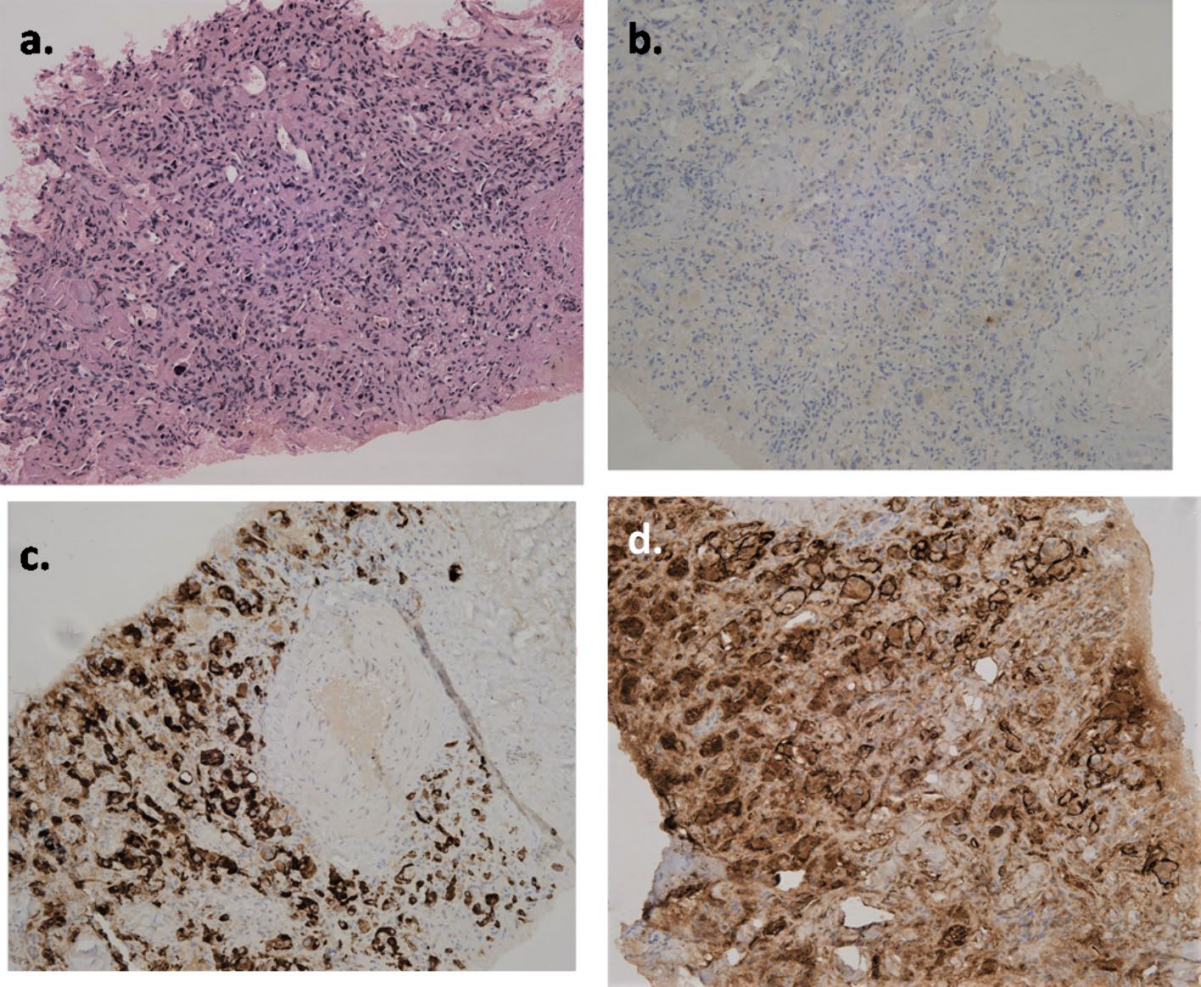
The core biopsy of the right carotid lesion (**a** H & E, 20×) shows nests of invasive tumour separated by thin fibrous septae. The tumour cells were medium sized with round nuclei, prominent nucleoli and moderate to abundant eosinophilic and granular cytoplasm. On immunohistochemistry, Pan-Cytokeratin **b** showed no expression in the tumour cells, however Synaptophysin **c** was positive and S100p **d** highlighted the sustentacular cells. This features were suggestive of carotid body paraganglioma

A 48 year old man presented with non-tender right sided neck swelling for last 20 years which was pulsatile in nature, freely movable in all directions and showed sudden increase in its size in last 1 year. This was associated with paraesthesia in both lower limbs and low back pain. There was no history of cranial nerve deficit, loss of weight and appetite or dysphagia. To rule out malignancy, F-18 FDG PET/CT was performed. As seen in Fig. 1, there was a metabolically active homogenously enhancing irregular mass (5 × 5.6 × 5.6 cm, SUV max- 21.2) in right carotid space causing splaying and encasement of the internal and external carotid arteries, with no separate visualization of these vessels in their middle 1/3rd course suggestive of carotid body tumor. Also noted similarly homogenously enhancing metabolically active two lesions each in segment IVa (1.2 × 1.3 cm, SUV max- 3.1) and VI (5 × 6.7 cm, SUV max- 5.3) of liver, as well as expansile mixed lytic sclerotic lesions in spinous process of D5 (SUV max- 3.2) and body of D9 (SUV max- 7) vertebrae suggestive of metastases. Figure 2 shows the histopathology and immunohistochemistry images of the right carotid mass that confirmed the diagnosis of carotid body paraganglioma.

## Discussion

Carotid body paraganglioma also known as Chemodectoma or glomus tumor, arise from the parasympathetic paraganglionic cells in the carotid body located on either side of the neck (Barnes et al. 2005). It is the most common head and neck paraganglioma, with characteristic presentation of splaying of the carotid arteries due to its location (Lack et al. 1977). However, as the tumor enlarges it may also result in the vascular encasement. It is usually benign and appear as a slow growing painless mass in the side of the neck. According to the WHO classification, all the extra-adrenal paraganglioma have malignant potential characterized by the presence of the paraganglionic tissue at the sites of non-paraganglionic cells in form of metastases (Lloyd et al. 2017). Carotid body paraganglioma is reported to have around 12% of malignant potential (Naik et al. 2013). Screening of this neuroendocrine tumor is done using Ultrasound and doppler study of the neck along with serum/urine catecholamines levels, while the Contrast enhanced Computed tomography (CECT) as well as Magnetic Resonance Imaging (MRI) are vital for its characterization, accurate detection and local extension owing to their high spatial resolution (Gimenez-Roqueplo et al. 2013; Berg et al. 2004). The functional imaging modalities are indicated for the initial staging as well as response assessment of the paragangliomas like other neuroendocrine tumors (Janssen et al. 2015, 2016; Archier et al. 2016; Han et al. 2019). The choice of the radiotracers for its assessment depend on the molecular classification of the paraganglioma owing to the different specific cell-receptor that are targeted. Functional imaging utilizes ligands that are specific to tumors and are attached to a radiotracer that can be detected, in order to optimize the sensitivity and specificity in identifying lesions. While Ga-68 SSTR PET/CT is documented to have high sensitivity and specificity in the detection and staging of head and neck paragangliomas (Han et al. 2019), F-18 FDG PET/CT has a role in metastatic evaluation of these tumors in two conditions. First, when the SSTR imaging is not available like in this case, and second when there is low SSTR expression in the tumor on Ga-68 PET/CT owing to de-differentiation indicating more aggressive lesion (Taïeb et al. 2019; Bozkurt et al. 2017; Timmers et al. 2012). In this case, the mutational analysis of the tumor and Ga-68 SSTR imaging were not done due to non-availability of the reagents. With this case, we emphasize on the vital role of F-18 FDG PET/CT in detection of metastases in carotid body paraganglioma.

## Data Availability

Not applicable.
